# Sentiment Analysis Methods for HPV Vaccines Related Tweets Based on Transfer Learning

**DOI:** 10.3390/healthcare8030307

**Published:** 2020-08-28

**Authors:** Li Zhang, Haimeng Fan, Chengxia Peng, Guozheng Rao, Qing Cong

**Affiliations:** 1 School of Economics and Management, Tianjin University of Science and Technology, Tianjin 300457, China; zhangli2006@tust.edu.cn (L.Z.); fanhaimeng_95825@163.com (H.F.); 2 College of Intelligence and Computing, Tianjin University, Tianjin 300350, China; pengchenxia@163.com (C.P.); chf@tju.edu.cn (Q.C.); 3 Tianjin Key Laboratory of Cognitive Computing and Applications, Tianjin University, Tianjin 300350, China; 4 School of New Media and Communication, Tianjin University, Tianjin 300072, China

**Keywords:** transfer learning, HPV vaccines, social media, ELMo, GPT, BERT

## Abstract

The widespread use of social media provides a large amount of data for public sentiment analysis. Based on social media data, researchers can study public opinions on human papillomavirus (HPV) vaccines on social media using machine learning-based approaches that will help us understand the reasons behind the low vaccine coverage. However, social media data is usually unannotated, and data annotation is costly. The lack of an abundant annotated dataset limits the application of deep learning methods in effectively training models. To tackle this problem, we propose three transfer learning approaches to analyze the public sentiment on HPV vaccines on Twitter. One was transferring static embeddings and embeddings from language models (ELMo) and then processing by bidirectional gated recurrent unit with attention (BiGRU-Att), called DWE-BiGRU-Att. The others were fine-tuning pre-trained models with limited annotated data, called fine-tuning generative pre-training (GPT) and fine-tuning bidirectional encoder representations from transformers (BERT). The fine-tuned GPT model was built on the pre-trained generative pre-training (GPT) model. The fine-tuned BERT model was constructed with BERT model. The experimental results on the HPV dataset demonstrated the efficacy of the three methods in the sentiment analysis of the HPV vaccination task. The experimental results on the HPV dataset demonstrated the efficacy of the methods in the sentiment analysis of the HPV vaccination task. The fine-tuned BERT model outperforms all other methods. It can help to find strategies to improve vaccine uptake.

## 1. Introduction

With the rapid development of social media, the public can share their emotion, opinion, medical experience, and professional knowledge on public health issues such as infectious disease prevention [1,2], drug safety supervision [3,4], health promotion [5,6,7], and vaccination [8,9,10,11].

Human papillomavirus (HPV) is the most widespread sexually transmitted infection (STI) around the world. It has been established that approximately 4% of all cancers are associated with HPV [1]. HPV vaccines can prevent most cancers and diseases caused by HPV infections [8]. Despite the recommendation about the vaccine’s safety and effect, HPV vaccination rates in many countries are still far lower than the goal set by Healthy People 2020 of 80% series completion for both adolescent males and females [9]. We need to explore the public sentiments towards HPV vaccination and then take corresponding measures to improve the vaccination rate further. Du et al. [10] collected and manually annotated 6000 tweets related to the HPV vaccine. Then, they constructed a hierarchical SVMs (support vector machines) and evaluated different feature combinations. Finally, they optimized the model parameters to maximize the model performance in analyzing public attitudes. Zhou et al. [11] used the connection information on social networks to improve the recognition of the negative emotions towards HPV vaccination.

However, most of these works were based on machine learning methods. These conventional methods cost significant time and labor on task-specific feature engineering [12]. Differently, deep learning methods can automatically extract features by unsupervised or semi-supervised learning algorithms [13]. Moreover, it can generate high-quality vector representations that differ from the low-quality vector representations generated by feature engineering [14]. However, the application of deep learning methods needs a large amount of annotated data. In some domains, such as public health, it is challenging to construct a large-scale annotated dataset because of the costly expense of data acquisition and annotation.

Transfer learning can solve the problem by leveraging knowledge obtained from a large-scare source domain to improve the classification performance in the target domain [15]. At its simplest, migrating pre-trained word vectors initializes the input of the deep learning model. The pre-trained word vectors obtained based on massive text data are an essential part of the learned semantic knowledge that can significantly improve natural language processing tasks based on deep learning. In natural language processing (NLP) tasks, there are several ways to employ transfer learning strategies. Generally, we can initialize input words by transferring pre-trained word embedding. The pre-trained word embeddings on large-scare corpus contain abundant syntactic and semantic knowledge, which significantly promotes the NLP tasks based on deep learning methods [16]. However, static word vectors such as Word2Vec only produce a fixed vector representation. They cannot solve the problem that the same word may have different meanings when it appears in different positions in the text. The emergence of deep neural networks allows language models to dynamically generate word vectors to solve the ambiguity of words in different situations.

With the emergence of pre-trained language models such as bidirectional encoder representations from transformers (BERT) [17], the model can generate dynamic word embeddings to tackle the polysemy. Recently, fine-tuning the pre-trained language model with limited annotated domain-specific data has achieved excellent performance in a series of NLP tasks [18]. Adhikari et al. [19] established stated-of-the-art results for four accessible datasets (Reuters, AAPD, IMDB, Yelp 2014) by fine-tuning BERT for document classification.

To find a transfer learning system that is able to extract comprehensive public sentiment on HPV vaccines on Twitter with satisfying performance, three transfer learning approaches were proposed to tackle the limitation of annotated data in the public health area. (i) One was separately transferring diverse word embeddings and then processing by bidirection gated recurrent unit with attention mechanism (BiGRU-Att), called DWE-BiGRU-Att (Diverse Word Embeddings Processed by BiGRU-Att). In this way, we could exploit the syntax and semantics in the pre-trained word embeddings to improve the deep learning model’s performance. (ii) As the static word embeddings could not solve the polysemy, we proposed the other two transferring learning methods. These two were fine-tuning pre-trained models with limited annotated data, called fine-tuning generative pre-training (GPT) and fine-tuning BERT.

## 2. Related Work

Nowadays, anyone with access to the Internet can express their opinions on various social media. Especially on public health issues such as infectious disease prevention, drug safety supervision, and vaccination, the public tends to post their medical experience or search for professional medical information online. Because of the public’s open participation, the information related to public health issues can be spread on the Internet in a fast way.

Many studies have analyzed public opinions based on social media data. Salathe et al. collected publicly available tweets during the outbreak of H1N1 influenza [20]. They manually annotated part of the collected tweets. Each tweet was annotated with negative, positive, or neutral sentiment towards influenza vaccination. Then, they trained a machine learning model with the labeled data. The model was used to classify the sentiment of the remaining unlabeled tweets automatically. Finally, they used the fully classified dataset to study the sentiment distribution of influenza vaccination.

Myslín et al. [5] used support vector machines, Naive Bayes, and k-Nearest Neighbors to analyze the public opinions towards tobacco and tobacco-related products based on Twitter data. Ginn et al. [21] manually annotated 10,822 tweets and then trained two machine learning models to monitor adverse drug reactions.

However, these works were mostly based on machine learning methods. These methods need sophisticated feature engineering. Moreover, the sparse vectors generated by feature engineering are inferior to the dense vectors generated by deep learning methods. However, high-quality, dense vectors need to be trained on a large corpus. In this way, we transferred the dense vectors pre-trained on the large-scare corpus to improve the deep learning model’s performance. Pre-trained dense vectors, containing learned syntax and semantics, can offer significant improvements over deep learning NLP tasks. Kim [22] initialized embeddings to pre-trained word vectors pre-trained on 100 billion words of Google News. Zhang et al. [23] treated multiple pre-trained word embeddings (Word2Vec, GloVe, and Syntactic embedding) as distinct groups and then applied convolutional neural networks (CNNs) independently to each group. The corresponding feature vectors (one per embedding) were then concatenated to form the final feature vector.

Transferring the learned semantics and syntax knowledge from the other missions has aroused a great interest in natural language processing (NLP) [24]. As an essential component of learned semantic knowledge, pre-trained word embeddings can offer significant improvements over deep learning NLP tasks. The generalization of word embeddings, sentence embeddings, or paragraph embeddings was also used as features in downstream missions like sentiment analysis, text classification, clustering, and translation [10]. Even though pre-trained word embeddings can improve the performance, the static word embeddings, such as Word2Vec, GloVe, and FastText [25], only produce fixed embedding and cannot solve the polysemy. With the emergence of deep neural networks, language models can yield dynamic word embedding to tackle the polysemy. McCann et al. [26] proposed contextualized word vectors (CoVe) by computing contextualized representations with neural machine translation encoder. Embeddings from language models (ELMo) [27] generated dynamic word embeddings by the concatenation of independently trained left-to-right and right-to-left long short-term memory networks (LSTM).

Bidirectional encoder representations from transformers (BERT) is a technique for NLP (natural language processing) pre-training developed by Jacob Devlin and his colleagues from Google [17]. The BERT model has achieved better performance in many sentiments analysis tasks of social media [28,29,30,31,32,33]. For example, in the work of Wang et al. [29], the BERT model was used to identify public negative sentiment categories in China regarding COVID-19 on Sina Weibo. In the work of Müller et al. [30], the COVID-Twitter-BERT model was a transformer-based model that pre-trained on a large corpus of Twitter messages on the topic of coronavirus disease 2019 (COVID-19). It outperformed the BERT-Large model on five different classification datasets. A Framework for twitter sentiment analysis based on BERT has been proposed in the work of Azzouza et al. [31]. The framework achieved high performance on the SemEval 2017 dataset. A knowledge enhanced BERT Model was proposed for depression and anorexia detection on social media in the work of [33].

In addition, the method of fine-tuning pre-trained language models has made a breakthrough in a series of NLP tasks. It can tackle the polysemy and only need a little annotated data to train the model. Howard et al. [18] proposed ULMFiT, the first universal method for text classification by the fine-tuning pre-trained language model. In the work of Biseda et al. [34], BERT models were fine-tuned for three pharmacovigilance of adverse drug reactions (ADRs) tasks and achieved high performance. Myagmar et al. [32] fine-tuned pre-trained language models of BERT and XLNet for the cross-domain sentiment classification. The experimental results showed that fine-tuning methods outperformed previous state-of-the-arts methods while exploiting up to 120 times fewer data.

## 3. Methods

In this section, we described in detail our three transfer learning approaches. One is transferring diverse word embeddings passed through BiGRU-Att (Section 3.1). The others are fine-tuning pre-trained models processed by a fully connected softmax layer (Section 3.2).

### 3.1. Diverse Word Embeddings Processed by BiGRU-Att

**Figure 1:**
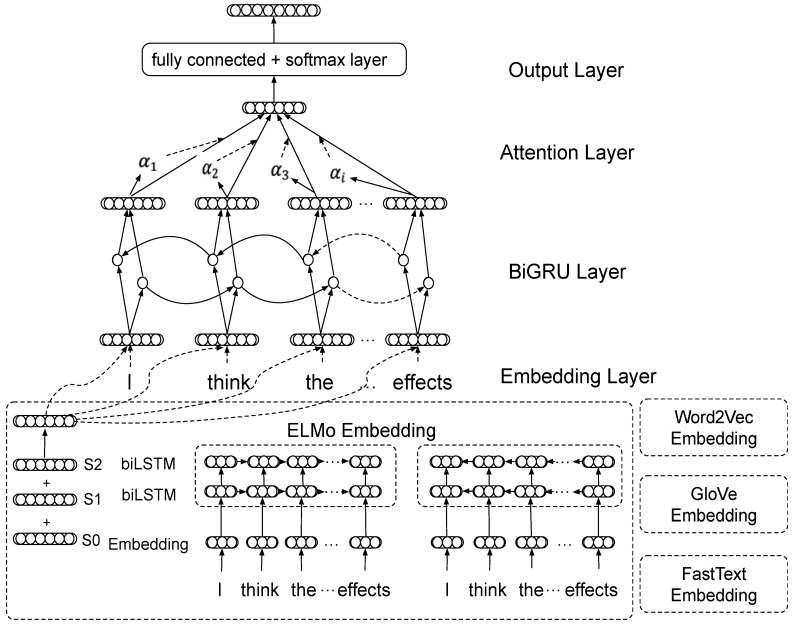
The architecture of diverse word embeddings processed by BiGRU-Att (DWE-BiGRU-Att).

We proposed the diverse word embeddings processed by BiGRU-Att (DWE-BiGRU-Att). Our four transfer learning methods are ELMo-BiGRU-Att, GloVe-BiGRU-Att, FastText-BiGRU-Att, and Word2Vec-BiGRU-Att. As shown in Figure 1, the architecture of our DWE-BiGRU-Att contains four components: embedding Layer, BiGRU Layer, attention Layer, and output Layer. For example, we took the sentence “I think the vaccine has side effects” as our method’s input.

#### 3.1.1. Embedding Layer

This layer maps each word into a dense dimension vector through transferring pre-trained word embedding. In this paper, we compared the results of static word embedding Word2Vec, GloVe, FastText, and contextualized word embedding ELMo.

The static word embeddings are separately 3 million 300-dimension Word2Vec word embedding trained on GoogleNews, 1 million 300-dimension FastText word embedding trained on Wikipedia, and 1.2 million 200-dimension GloVe word embedding trained on Twitter. If the word is concluded in the pre-trained embedding, we can get the word vector directly. If not, we generate the word vector randomly.

Deep contextualized word embeddings supposed by language model ELMo improve word representation quality and handle the polysemy problem to a certain extent. Different from the static word embeddings, it represents a word according to its context.

ELMo embedding is a combination of multiple layer representations in the bidirectional language model (biLM). Language model (LM) is the maximum likelihood of multiple sequences of K tokens, ($t_{1},t_{2},\ldots ,t_{K}$). The forward LM computes the probability of the next word $t_{n}$ given the history ($t_{1},t_{2},\ldots ,t_{n-1}$).

(1) $$p\left(t_{1},t_{2},\ldots ,t_{N}\right)=\prod_{n=1}^{K}p(t_{n}|t_{1},t_{2},\ldots ,t_{n-1})$$

Similarly, a backward LM predicts the before token based on the future context.

(2) $$p\left(t_{1},t_{2},\ldots ,t_{N}\right)=\prod_{n=1}^{K}p(t_{n}|t_{n+1},t_{n+2},\ldots ,t_{K})$$

A biLM combines the forward LM and backward LM and then maximizes the log-likelihood of the forward and backward LM. $\Theta_{x}$ and $\Theta_{s}$ are respectively the token representation and softmax parameters, which are shared in the forward and backward directions. ${\vec{\Theta }}_{LSTM}$ and ${\overset{\leftarrow }{\Theta }}_{LSTM}$ are the parameters of biLM.

(3) $$biLM=\sum_{n=1}^{K}(logp\left(t_{n}|t_{1},\ldots ,t_{n-1};\Theta_{x},{\vec{\Theta }}_{LSTM},\Theta_{s}\right)+logp(t_{n}|t_{n+1},\ldots ,t_{K};\Theta_{x},{\overset{\leftarrow }{\Theta }}_{LSTM},\Theta_{s}))$$

For each token $t_{n}$, a L-layer biLM computes a set of 2L+1 representations:

(4) $$R_{n}=\{x_{n}^{LM},{\vec{h}}_{n,j}^{LM},{\overset{\leftarrow }{h}}_{n,j}^{LM}\;|\;j=1,\ldots ,L\}=\{h_{n,j}^{LM}\;|\;j=0,\ldots ,L\}$$

$h_{n,j}^{LM}$ is calculated by $h_{n,j}^{LM}=\left[{\vec{h}}_{n,j}^{LM};{\overset{\leftarrow }{h}}_{n,j}^{LM}\right]$ for each biLSTM layer. ELMo integrates the output $R_{n}$ of multilayer biLM into a single vector, $ELMo_{n}=E\left(R_{n},\Theta_{e}\right)$. The simplest case is that ELMo uses only the topmost output, $E\left(R_{n}\right)=h_{n,j}^{LM}$. Here, our ELMo adds the output of all biLM layer multiplied by the softmax-normalized weights $s^{task}$. $\gamma^{task}$ is a hyperparameter for optimization and scaling the ELMo vector.

(5) $$ELMo_{n}^{task}=E\left(R_{n};\Theta^{task}\right)=\gamma^{task}\sum_{j=0}^{L}s_{j}^{task}h_{n,j}^{LM}$$

#### 3.1.2. BiGRU Layer

This layer is built to aggregate the word representations containing the bidirectional information. The BiGRU layer takes the dense word embeddings $V\in R_{t\times d}$ as input. t is the number of words in the input context and d is the dimension of the word vector. The BiGRU layer consists of two GRU layers that process the information from both forward GRU neuron and backward GRU neuron.

**Figure 2:**
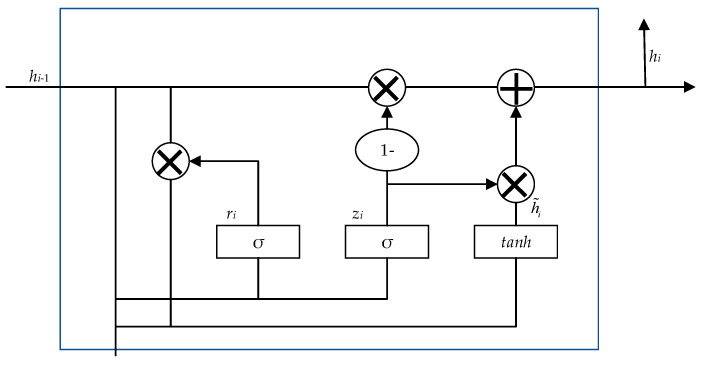
The architecture of the gated recurrent unit (GRU).

Figure 2 shows the structure of the cell unit in the GRU. Two new gates $r_{i}$ and $z_{i}$ are added to the cell unit to solve the gradient disappearance problem of standard RNN. $r_{i}$ determines how much of the past information needs to be retained, and $z_{i}$ helps the model determine how much of the past information needs to be passed to the candidate hidden state. The calculation process of the reset gate $r_{i}$ is as follows:

(6) $$r_{i}=\sigma \left(W^{r}x_{i}+U^{r}h_{i-1}\right)$$

where σ is the activation function, $x_{i}$ is the input, $h_{i-1}$ is the hidden state of the previous cell unit, and $W^{r}$ and $U^{r}$ are the weight matrix.

Similarly, the update gate $z_{i}$ is calculated as follows:

(7) $$z_{i}=\sigma \left(W^{z}x_{i}+U^{z}h_{i-1}\right)$$

Formally, the formula of current hidden state $h_{i}$ can be formalized as

(8) $$h_{t}=\left(1-z_{t}\right)\times h_{t-1}+z_{t}\times \tilde{h_{t}}$$

The formula for calculating $\tilde{h_{t}}$ is as follows:

(9) $$\tilde{h_{t}}=\tan h\left(Wx_{t}+r_{t}UWh_{t-1}\right)$$

The forward GRU extracts the word feature as $\vec{h_{l}}$, and the backward GRU extracts the feature as $\overset{\leftarrow }{h_{l}}$. The resulting hidden states of each GRU cell for both directions $\vec{h_{l}}$ and $\overset{\leftarrow }{h_{l}}$ are concatenated together for each time step i=1…t. The t is the number of input tokens. Then, we obtain the final sequence of word features $H=\left(h_{1},h_{2},h_{i},\ldots ,h_{n}\right)$ where $h_{i}$ is calculated by $h_{i}$= [$\vec{h_{l}},\;\overset{\leftarrow }{h_{l}}$]. $h_{i}$ concatenates the bidirectional information to summarize the information of the whole context centered around the word.

#### 3.1.3. Attention Layer

Because not all words make the same contribution in understanding the sentence’s meaning, we employed the attention mechanism to implement the contribution of important words.

The resulted concatenation of the representations of the forward and backward GRU, $h_{i}=\left[\vec{h_{l}}\;,\;\overset{\leftarrow }{h_{l}}\right]$, is then converted to Formula (10) through a fully connected layer.

(10) $$u_{i}=\tan h\left(W_{w}h_{i}+b_{w}\right)$$

Then, the probability distribution $\alpha_{i}$, representing the importance of each sentence in the context, is obtained by calculating the similarity between $u_{i}$ and the context vector $u_{w}$ and softmax operation.

(11) $$\alpha_{i}=\frac{exp\left(u_{i}u_{w}\right)}{\sum_{t}exp\left(u_{i}u_{w}\right)},\;\sum_{i=1}^{t}\alpha_{i}=1$$

At last, the document representation $s_{i}$ is the weighted sum of $\alpha_{i}$ and $h_{i}$.

(12) $$s_{i}=\sum_{t}\alpha_{i}h_{i}$$

#### 3.1.4. Output Layer

**Figure 3:**
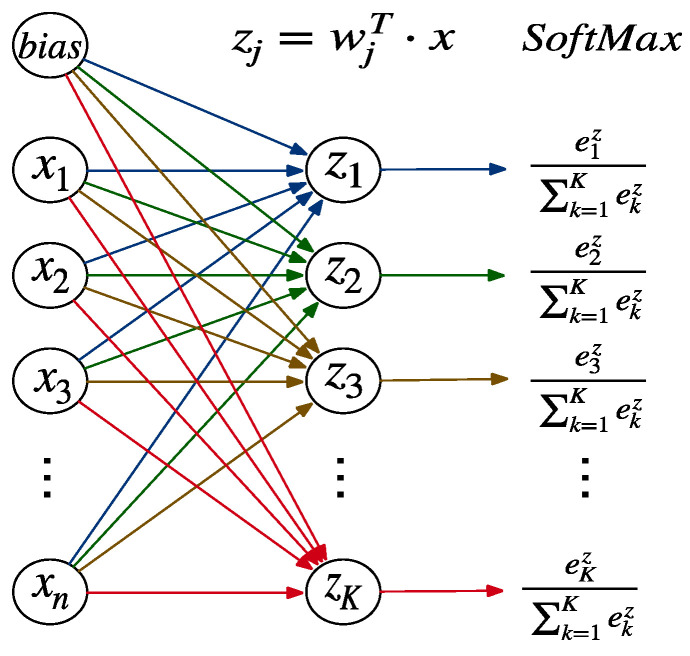
Multi-class fully connected and Softmax layers.

The vector representation of the input text generated by the attention layer represents the probability distribution that $s_{i}$ gets the public’s opinion labels on public health issues through the fully connected Softmax layer. Figure 3 shows the multi-class fully connected and Softmax layers corresponding to the output layer. The function of the fully connected and Softmax layer is to map the 𝑛 dimension vector composed of 𝑛 real numbers between negative infinity to positive infinity into the 𝐾 dimension vector composed of 𝐾 real numbers between 0 and 1. Moreover, the sum of 𝐾 real numbers is equal to 1. The calculation process is shown in Formula (13).

(13) $$\hat{y}=softmax\left(z\right)=softmax\left(W^{T}x+b\right)$$

The Softmax is calculated as follows:

(14) $$softmax\left(z_{j}\right)=\frac{e^{z_{j}}}{\sum_{K}e^{z_{j}}}$$

The specific probability of each category is calculated as follows, where $w_{j}$ represents the weight vector composed of the same color in the Figure 3.

(15) $$\hat{y_{j}}=softmax\left(z_{j}\right)=softmax\left(w_{j}\cdot x+b_{j}\right)$$

The representation $s_{i}$ generated from the attention layer is fed into a fully connected softmax layer to obtain the distribution of class probability. We minimized categorical cross-entropy loss function J in which loss increases as the $i_{th}$ predicted probability $p_{i}$ deviates from the actual label $y_{i}$. the loss function J is calculated as follows:

(16) $$J=-\sum_{i=1}^{K}y_{i}\log\left(p_{i}\right)$$

### 3.2. Fine-Tuning Pre-trained Models

Although transferring word embeddings can offer significant improvements in many NLP tasks, it is more efficient to fine-tune pre-trained language models with a little labeled target-domain data. In this section, we respectively described our fine-tuned GPT and fine-tuned BERT public sentiment analysis classifier.

#### 3.2.1. Fine-Tuning GPT

**Figure 4:**
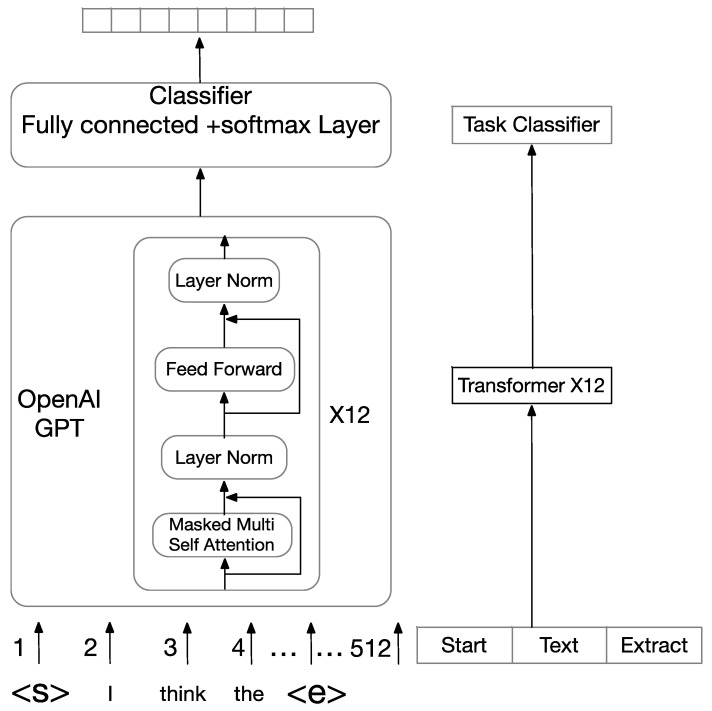
The architecture of fine-tuning generative pre-training (GPT).

In Figure 4, GPT uses multi-layer transformer decoders as a feature extractor. The transformer decoders are more powerful than the LSTM in handling long-term dependency. Our fine-tuned GPT public sentiment analysis classifier must apply the same structure as GPT pre-training. We also need to process the input context differently.

We assumed a labeled dataset C in which each case contains a sequence of words, ($x^{1},\ldots ,x^{n}$), along with a label y. For our classification task, our inputs need to add randomly initialized start and end tokens (<s>, <e>). The pre-trained GPT model processes the recombined inputs. Then, we obtained $h_{l}^{n}$, which was the output of the final transformer block. The $h_{l}^{n}$ is then fed into a fully connected softmax layer with matrix $W_{y}$ to predict y.

(17) $$P\left(y|x^{1},\ldots ,x^{n}\right)=softmax\left(h_{l}^{n}W_{y}\right)$$

Lastly, we got the optimization objective to maximize:

(18) $$L_{2}\left(C\right)=\sum_{\left(x,y\right)}logP(y|x^{1},\ldots ,x^{n})$$

#### 3.2.2. Fine-Tuning BERT

**Figure 5:**
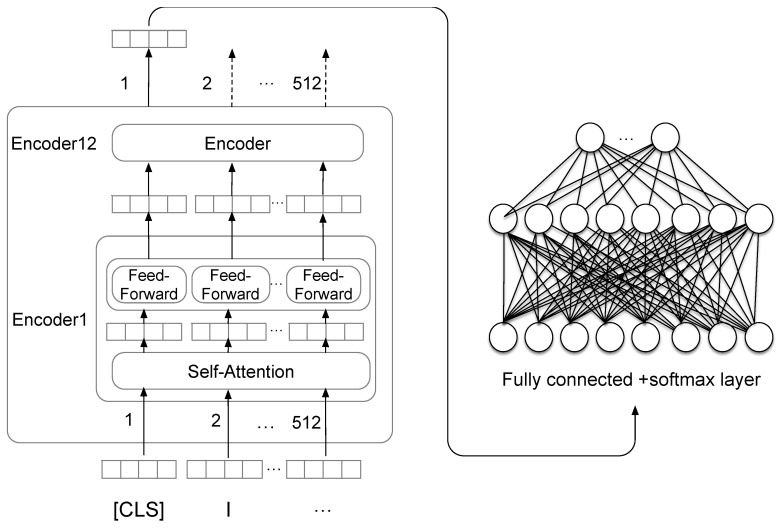
The architecture of fine-tuning bidirectional encoder representations from transformers (BERT).

Unlike GPT employing a left-to-right transformer, BERT utilizes a bidirectional transformer. In this paper, we fine-tuned BERTbase. It contains 12 transformer blocks, 12 self-attention heads, and 768 hidden units. As seen in Figure 5, BERT base takes a sequence of no more than 512 tokens as input and outputs the representation of the sequence.

In our classification task, BERT base takes the final hidden state $C\in R^{H}$ of the first token [CLS] as the representation of the whole sequence. We introduced a fully connected softmax layer over the final hidden state C. The softmax classifier parameter matrix is $W\in R^{K\times H}$, where H is the dimension of the hidden state vectors and K is the number of classes.

(19) $$P=softmax\left(CW^{T}\right)$$

We minimized the categorical cross-entropy loss and fine-tune all the parameters from BERT as well as W to maximize the probability of the correct label.

## 4. Experiments and Results

### 4.1. Data Source and Data Processing

#### 4.1.1. Data Source

Experiments were conducted on 6000 annotated HPV-related tweets [10]. The combinations of keywords (HPV, human papillomavirus, Gardasil, and Cervarix) are used to collect public tweets using the official Twitter application programming interface (API) [35]. 33,228 English tweets containing HPV vaccines related keywords in total were collected from 15 July 2015 to 17 August 2015. Then, the URLs and duplicate tweets were removed. 6000 tweets were selected for annotation randomly. In Table 1, we divided the dataset into eight categories through the hierarchical structure. Then, each tweet had one category label. The hierarchical structure was based on the subdivision of unbalanced data. First, according to whether the tweet was related to HPV or not, we divided the tweet into a related class or unrelated class. Next, the tweets that belong to the related class were divided into positive class, neutral class, and negative class. Last, the negative tweets were classified into NegSafety class, NegEfficacy class, NegResistant class, NegCost class, and NegOthers class based on some most common worries about the vaccination like side effects, efficacy, cost, and culture-related issues. The detailed proportion of each category is shown in Table 1.

#### 4.1.2. Data Processing

There were some essential data cleaning and pre-processing work we had done, including lowercase letter replacement, deleting punctuation, excluding hashtags, user names (e.g., @user), and replacing all URLs (e.g., ‘http://xx.com’) with ‘URL’. Table 2 showed two processed sentences.

### 4.2. Experimental Setup

We applied 10-fold cross-validation to make full use of the small dataset and ensure the same evaluation indicators with the work of [10]. So, leave one out cross-validation is not applied in this paper. For each category, we treated it as a binary classification and assessed consequence with the $F_{1}$-score. The $F_{1}$-score is defined as the harmonic mean of the precision and recall of a binary decision rule [36]. For overall performance, we used micro-*F*_1_ as multiclass classification assessment indexes. The Formula (20) showed the specific calculation process:

(20) $$\mathrm{Micro}\_F_{1}=\frac{2\times Micro\_P\times Micro\_R}{Micro\_P+Micro\_R}\;,\;\mathrm{Micro}\_P=\frac{\sum_{i=1}^{m}TP_{i}}{\sum_{i=1}^{m}TP_{i}+\sum_{i=1}^{m}FP_{i}}\;,\;\mathrm{Micro}\_R=\frac{\sum_{i=1}^{m}TP_{i}}{\sum_{i=1}^{m}TP_{i}+\sum_{i=1}^{m}FN_{i}}$$

$\mathrm{Micro}\_F_{1}$ calculates the proportion of instances predicted correctly in the predicted samples (regardless of the category) with Formula (20) where Micro_P is micro-average of precision, Micro_R is micro-average of recall, $TP_{i}$ is the true positive sample, $FP_{i}$ is the false positive sample, and $FN_{i}$ is false negative sample.

The optimal parameter settings are given in Table 3.

### 4.3. Baselines

We compared our transfer learning methods with traditional machine learning models, including plain support vector machines (SVMs) and hierarchical SVMs, and general deep learning models (i.e., attention-based BiGRU model [37]). The plain SVM classification used word-ngrams as features and chose default SVMs parameters. The hierarchical SVMs used three SVMs models trained independently and chose word-ngrams as features. The results of these models came from [10].

### 4.4. Results

#### 4.4.1. Average of Micro Index

The micro-average can be a useful measure when your dataset varies in small size. In Table 4, The 10-fold cross-validation performance of the average of micro index on the baseline models (plain SVM and hierarchical SVMs and BOW-BiGRU-Att) and our transfer learning models are shown. The plain SVM classification results used word-ngrams as the feature and chose default SVMs parameters and the hierarchical SVMs that used three SVMs models trained independently and chose word-ngrams as the feature are the official numbers from [10]. The columns of BOW-BiGRU-Att, Word2Vec-BiGRU-Att, FastText-BiGRU-Att, GloVe-BiGRU-Att, and ELMo-BiGRU-Att are our experiment results of bidirectional long short-term memory combined with bag-of-word, Word2Vec, pre-trained FastTest embedding, GloVe embedding, and ELMo embedding respectively. The columns of FT-GPT-FC and FT-BERT-FC are the results of fine-tuning GPT and fine-tuning BERT models, respectively. FT-GPT-FC and FT-BERT-FC respectively represent the fine-tuned model with a fully connected neural network. The row of Average/Method represents the average of micro-*F*_1_ score of 10-fold cross-validation on each method. The column of Average/Fold represents the average of micro-*F*_1_ score of each fold on all methods. The FT-BERT-FC gets the best performance with the bold number. The result of FT-BERT-FC is 0.769. It makes 14.8% and 6.95% increase than the plain SVM and the hierarchical SVMs score (0.670 and 0.719), respectively. The ELMo-BiGRU-Att and FT-GPT-FC also increase by 2.68% and 1.53% more than hierarchical SVMs on micro-*F*_1_ average, respectively. The better performance of FT-BERT-FC can be attributed to the fact that the left-to-right and right-to-left transformers of BERT is more powerful than the left-to-right transformer of GPT. The bidirectional transformer concentrates on the left and right context of the word, but the left-to-right transformer can only focus on the left context of the word. Thus, pre-trained BERT can extract more high-quality feature vectors. The other results are 0.654, 0.697, 0.708, and 0.702 are all lower than hierarchical SVMs. However, the performance of all except the BOW-BiGRU-Att is better than plain SVM.

Among all DWE-BiGRU-Att models (ELMo-BiGRU-Att, GloVe-BiGRU-Att, FastText-BiGRU-Att, and Word2Vec-BiGRU-Att), ELMo-BiGRU-Att obtain the highest micro-$F_{1}$ average. The results indicate that dynamic word embedding (ELMo) is more efficient than static word embeddings (GloVe, FastText, and Word2Vec). Meantime, ELMo can solve the polysemy that cannot be handled by static word embeddings. However, the $\mathrm{Micro}-F_{1}$ average of FT-GPT-FC are increased by 0.82% than ELMo-BiGRU-Att.

Compared with BOW-BiGRU-Att, the micro-$F_{1}$ average of Word2Vec-BiGRU-Att is still increased by 6.57%. The significant improvement means that transferring pre-trained word embedding is efficient in promoting the classification performance of deep learning methods.

#### 4.4.2. Standard Deviation and Root Mean Square Error

The standard deviation (SD) of the micro-*F*_1_ score for all methods is given in the row of SD to measure the variance of a model’s performance. Root mean square error (RMSE) is applied as an error analysis. The RMSE is calculated as follows where m is the sample size, $y_{\mathrm{test}}^{\left(i\right)}$ is observed values, ${\hat{y}}_{\mathrm{test}}^{\left(i\right)}$ is expected values.

(21) $$RMSE=\sqrt{\frac{1}{m}\sum_{i=1}^{m}{\left(y_{test}^{\left(i\right)}-{\hat{y}}_{test}^{\left(i\right)}\right)}^{2}}$$

The SD and RMSE are shown in Table 5. The SD and RMSE of FT-BERT-FC are lower than the values of the plain SVM and hierarchical SVMs in average of micro index. The performance of the plain SVM and hierarchical SVMs depend on the feature extracting from the training data set, so the performance of each fold is more different. The SD and RMSE of the dynamic embeddings model (such as FT-BERT-FC and FT-GPT-FC) are lower than that of the static embeddings model (such as FastText-BiGRU-Att and GloVe-BiGRU-Att). Generally, the static embeddings model requires much larger amounts of data. They can get major improvements when trained on millions or more annotated training examples. However, the BERT model trained general-purpose language representation models using the enormous piles of unannotated text on the web (this is known as pre-training). The FT-BERT-FC method is not needed to extract high-quality language features from the text data, we fine-tuned the model with BERT on the HPV vaccination task to produce state-of-the-art predictions.

## 5. Discussion

### 5.1. Micro-$F_{1}$ Scores in Each Fold

Table 4 shows the specific micro-*F*_1_ scores of different models in each fold (F-1, F-2, …, F-10). We sum up the true positives (TP), false positives (FP), and false negatives (FN) of the system for different sets and apply them to get the statistics. The bold number denotes the largest number in that row. In all folds, FT-BERT-FC gains the highest scores all, which indicates the robustness of the model. The micro-*F*_1_ score of FT-GPT-FC and ELMo-BiGRU-Att are relatively close in each fold. The difference between their scores of each fold does not exceed 0.02. Furthermore, the performance of Word2Vec-BiGRU-Att, FastText-BiGRU-Att, and GloVe-BiGRU-Att is similar in each fold. It indicates that the Word2Vec, FastText, and GloVe.embedding mechanisms have similar effects on the HPV dataset.

The worst overall performance of all methods emerges in the third fold F-3, which means the overall micro index performance of all models is the worst. The average micro-*F*_1_ score of the third ford is 0.686. Correspondingly, the highest average micro-*F*_1_ score of each ford is 0.732 in the fourth fold F-4. That means the overall micro index performance of all models is the best in this fold.

### 5.2. Statistical Test

We chose the Friedman test with the Nemenyi post hoc test based on [38]. The Friedman test is a non-parametric statistical test developed by Milton Friedman [39]. It can be used to detect differences in multiple methods across multiple test data sets. The steps of the Friedman test and the Nemenyi test for this paper are given as follows.

(1) Define Null and Alternative Hypotheses

H_0_: There is no difference between the nine methods; H_1_: There is a difference between the nine methods.

(2) Calculate Test Statistic

First, from Table 5, We ranked the methods for each fold (F-1, F-2, …, F-10) separately on micro-$F_{1}$ score. Second, we replaced our original values with the rankings as shown in Table 6. Let $r_{i}^{j}$ be the rank of the j−th of k methods on the i−th of N fold. The Friedman test compares the average ranks (mean ranks) of methods, $R_{j}=\frac{1}{N}\overset{N}{\underset{i=1}{\sum }}r_{i}^{j}$. The Friedman statistic is distributed according to $\chi_{F}^{2}$ with k − 1 degrees of freedom.

(22) $$\chi_{F}^{2}=\frac{12N}{k\left(k+1\right)}\left[\sum_{j=1}^{k}R_{j}{}^{2}-\frac{k{\left(k+1\right)}^{2}}{4}\right]$$

Friedman’s $\chi_{F}^{2}$ is undesirably conservative and derived a better statistic was proposed by Iman and Davenport [40].

(23) $$F_{F}=\frac{\left(N-1\right)\chi_{F}^{2}}{N\left(k-1\right)-\chi_{F}^{2}}$$

$F_{F}$ is distributed according to the F-distribution with *k* − 1 and (*k* − 1) (*N* − 1) degrees of freedom. The table of critical values can be found in any statistical book. In this paper, N=10, k=9. With nine algorithms and 10-fold cross-validation data sets, $F_{F}$ is distributed according to the F distribution with 9 − 1 = 8 and (9 − 1) × (10 − 1) = 72 degrees of freedom. The critical value of *F* (8,72) for α = 0.05 is 2.07. We got $\chi_{F}^{2}=73.57$, $F_{F}=103.03$ with Equations (23) and (24). $F_{F}>F_{0.05}\left(8,\;72\right)$ where α=0.05. So, we reject the null hypothesis. We proceed with a post hoc test using the Nemenyi test [41].

(3) Nemenyi test

The performance of different methods is significantly different if the corresponding average ranks differ by at least the critical difference (*CD*).

(24) $$CD=q_{\alpha }\sqrt{\frac{k\left(k+1\right)}{6N}}$$

At *p*-value = 0.05, $q_{0.05}=3.102$ were obtained from a *F*-distribution table in any statistical book where α=0.05. Then, CD is 3.80 calculated with Equation (25).

(25) $$CD=3.102\times \sqrt{\frac{9\times 10}{6\times 10}}=3.80$$

**Figure 6:**
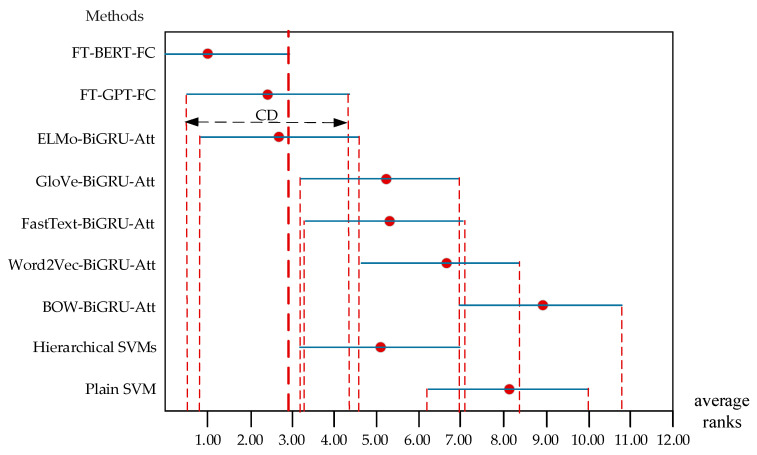
Comparison of all methods against each other with Nemenyi test.

All the $R_{j}\pm \frac{CD}{2}$ were got and shown in Table 6. The critical difference (CD) diagrams are shown in Figure 6. We can identify the performance of FT-BERT-FC is significantly better than that of plain SVM [10], hierarchical SVMs [10], BOW-BiGRU-Att, Word2Vec-BiGRU-Att, FastText-BiGRU-Att, and GloVe-BiGRU-Att. We cannot tell that there is a significant difference between FT-BERT-FC, ELMo-BiGRU-Att, and FT-GPT-FC. We can conclude that the post hoc test is not powerful enough to detect any significant differences between the ELMo-BiGRU-Att, FT-GPT-FC, and hierarchical SVMs at *p*-value is equal to 0.05.

### 5.3. Limitations and Future Researches

We have demonstrated how the methods of the sentiment analysis of the HPV vaccination task. However, only one dataset with 6000 tweets is verified. One of the next steps is to study the performance of these methods working on different sizes and multi-domain.

The plain SVM, hierarchical SVMs, BOW-BiGRU-Att, Word2Vec-BiGRU-Att, FastText-BiGRU-Att, GloVe-BiGRU-Att, and ELMo-BiGRU-Att are based on annotated Twitter data whereas FT-GPT-FC and FT-BERT-FC are not. However, FT-GPT-FC and FT-BERT-FC are pre-trained models, so they need more high-performance computing resources to conduct experiments.

Some tweets are not be processed by FT-BERT-FC shown in Table 7. The current methods usually neglect to consider commonsense knowledge for public opinions on public health issues. Knowledge enhanced ensemble learning models on social media should be tried to address this problem.

Furthermore, uneven data distribution is an excellent challenge for the current models. There are only 6 NegResistant and 6 NegCost tweets in the dataset. Some deep learning approaches for processing imbalanced data should be studied as [7].

## 6. Conclusions

We try to find a transfer learning system that can extract comprehensive public sentiment on HPV vaccines on Twitter with satisfying performance. We proposed three transfer learning approaches to analyze public sentiments towards public health issues for the goal. To exploit syntax and semantics pre-trained on a large corpus, a method of transferring diverse word embeddings was combined with BiGRU-Att layer. As the static word embeddings could not solve the polysemy, we proposed the other two methods of fine-tuning GPT and fine-tuning BERT. In this way, we could take advantage of the strong feature extraction capability of large neural networks by using a little annotated target-domain data to fine-tune the language model. The experimental results showed the superiority of FT-BERT-FC for the HPV vaccination issue. With the success of this work, our transfer learning approaches were expected to be further applied to other public sentiments tasks towards public health issues.

## Figures and Tables

**Table 1:** The detailed proportion of each category.

Category	Topic (HPV)	Sentiment	Sentiment (Subclass)	Tweet Numbers (Proportion)	Example
1	Unrelated	/	/	2016 (33.6%)	Only three U.S. states mandate recommended HPV vaccine http://t.co/YCInira89m via @Reuters
2	Related	Positive	/	1153 (19.2%)	RT @GlowHQ: Dear #HPV Vaccination. You are safe & effective. Why don’t more states require you? @VICE http://t.co/QRL26SA4GO http://t.co/gY.
3		Neutral	/	1386 (23.1%)	Gardasil HPV Vaccine Safety Assessed In Most Comprehensive Study To Date http://t.co/4g3ztZdSU4 via @forbes.
4		Negative	NegSafety	912 (15.2%)	Worries about HPV vaccine: European Union medicines agency investigating reports of rar http://t.co/bMOr3XveVC http://t.co/jZeHFkCDpl.
5			NegEfficacy	46 (0.77%)	ACOG is now “recommending” ob/gyn’s to push HPV vaccine despite its ineffectiveness & it’s notorious track record of killing &maiming ppl.
6			NegResistant	6 (0.1%)	#HPVvaccine “would introduce sexual activity in young women, that would inappropriately introduce promiscuity” http://t.co/zEnDdyVP8a.
7			NegCost	6 (0.1%)	RT @kylekirkup: I’m no public health expert, but huh?! If you’re male & want free HPV vax in BC, you have to come out. At age 11. http://.
8			NegOthers	475 (7.93%)	Sanofi Sued in France over Gardasil #HPV #Vaccine –http://t.co/LruYf4c0co.

**Table 2:** The process of data cleaning.

Unprocessed Tweets	Processed Tweets
@margin What’s your attitude about the vaccination? https://stamp.jsp?tp=&arnumber=897 Please write me back @Daviadaxa soon!!!!! http://#view=home&op=translate&sl=auto	What’s your attitude about the vaccination url please write me back soon url

**Table 3:** The values of all parameters.

Parameter	Value
Loss Function	Categorical cross-entropy
Train-Test Split	10-fold cross-validation
Optimizer	Adam
Learning Rate	0.001
Back-Propagation	ReLu
Batch Size	32
Dropout	0.25
Hidden State GRU	64

**Table 4:** The 10-fold cross-validation Micro-$F_{1}$ score on all methods.

Fold	Methods-Other Teams		Methods-Our Works							Average/Fold
	Plain SVM [10]	Hierarchical SVMs [10]	BOW-BiGRU-Att	Word2Vec-BiGRU-Att	FastText-BiGRU-Att	GloVe-BiGRU-Att	ELMo-BiGRU-Att	FT-GPT-FC	FT-BERT-FC	
**F-1**	0.682	0.739	0.658	0.710	0.728	0.719	0.750	0.743	0.789^1^	0.724
**F-2**	0.671	0.698	0.650	0.699	0.701	0.704	0.724	0.722	0.755	0.702
**F-3**	0.639	0.682	0.643	0.677	0.673	0.680	0.707	0.721	0.750	0.686
**F-4**	0.693	0.743	0.669	0.724	0.737	0.727	0.745	0.768	0.778	0.732
**F-5**	0.658	0.721	0.645	0.681	0.712	0.691	0.722	0.730	0.762	0.702
**F-6**	0.677	0.728	0.662	0.700	0.680	0.703	0.731	0.735	0.771	0.710
**F-7**	0.642	0.690	0.631	0.686	0.719	0.695	0.712	0.721	0.753	0.694
**F-8**	0.669	0.729	0.660	0.712	0.723	0.719	0.736	0.744	0.776	0.719
**F-9**	0.690	0.735	0.668	0.703	0.718	0.702	0.749	0.747	0.791	0.723
**F-10**	0.678	0.723	0.649	0.681	0.691	0.677	0.721	0.730	0.762	0.701
**Average/Method**	0.670	0.719	0.654	0.697	0.708	0.702	0.730	0.736	0.769	/

^1^ The FT-BERT-FC gets the best performance with the bold number in each fold.

**Table 5:** Standard deviation and root mean square error.

Research Team	Methods	SD	RMSE
Other Teams	Plain SVM [10]	0.018	0.017
	Hierarchical SVMs [10]	0.022	0.021
Our Works	BOW-BiGRU-Att	0.013	0.012
	Word2Vec-BiGRU-Att	0.014	0.013
	FastText-BiGRU-Att	0.023	0.021
	GloVe-BiGRU-Att	0.017	0.016
	ELMo-BiGRU-Att	0.016	0.015
	FT-GPT-FC	0.016	0.015
	FT-BERT-FC	0.015	0.014

**Table 6:** The 10-fold cross-validation rank of Micro-$F_{1}$ score.

Fold	Methods-Other Teams		Methods-Our Works						
	Plain SVM [10]	Hierarchical SVMs [10]	BOW-BiGRU-Att	Word2Vec-BiGRU-Att	FastText-BiGRU-Att	GloVe-BiGRU-Att	ELMo-BiGRU-Att	FT-GPT-FC	FT-BERT-FC
**F-1**	8	4	9	7	5	6	2	3	1
**F-2**	8	7	9	6	5	4	2	3	1
**F-3**	9	4	8	6	7	5	3	2	1
**F-4**	8	4	9	7	5	6	3	2	1
**F-5**	8	7	9	6	5	4	2	3	1
**F-6**	8	4	9	7	5	6	3	2	1
**F-7**	8	4	9	6	7	5	3	2	1
**F-8**	8	6	9	7	3	5	4	2	1
**F-9**	8	7	9	6	5	4	2	3	1
**F-10**	8	4	9	7	5	6	3	2	1
$R_{j}$	8.1	5.1	8.9	6.5	5.2	5.1	2.7	2.4	1.0
$R_{j}{}^{2}$	65.61	26.01	79.21	42.25	27.04	26.01	7.29	5.76	1.00
$R_{j}\;\pm \;\frac{CD}{2}$	8.1 ± 1.90	5.1 ± 1.90	8.9 ± 1.90	6.5 ± 1.90	5.2 ± 1.90	5.1 ± 1.90	2.7 ± 1.90	2.4 ± 1.90	1.0 ± 1.90

**Table 7:** Some tweets are not processed correctly by FT-BERT-FC.

No.	Tweet	Annotated Category	The Category Identified by FT-BERT-FC
1	Warts are cause by HPV	Unrelated	Neutral
2	@handronicus she is not pleased with me. She hasn’t been this mad since I got the cervical cancer vaccine (only sluts get HPV duh)	NegResistant	NegOthers
3	RT @kylekirkup: I’m no public health expert, but huh?! If you’re male & want free HPV vax in BC, you have to come out. At age 11. http://…	NegCost	NegSafety
	…	…	…
